# Low tuberculosis notification in mountainous Vietnam is not due to low case detection: a cross-sectional survey

**DOI:** 10.1186/1471-2334-7-109

**Published:** 2007-09-19

**Authors:** M Vree, NB Hoa, DN Sy, NV Co, FGJ Cobelens, MW Borgdorff

**Affiliations:** 1 KNCV Tuberculosis Foundation, The Hague, The Netherlands; 2 Center for Infection and Immunity Amsterdam (CINIMA), Academic Medical Center, Amsterdam, The Netherlands; 3 National Tuberculosis Programme Vietnam, Hanoi, Vietnam

## Abstract

**Background:**

Studies show that tuberculosis notification declines with increasing altitude. This can be due to declining incidence or declining case detection. In Vietnam notification rates of new smear-positive tuberculosis in the central mountainous provinces (26/100,000 population) are considerably lower than in Vietnam in general (69/100,000 population). In order to clarify whether this is explained by low incidence or low case detection, we aimed to assess the prevalence of new smear-positive tuberculosis among adults with prolonged cough in three mountainous provinces in central Vietnam.

**Methods:**

A house-to-house survey of persons (≥ 15 years) was carried out in twelve randomly selected districts in 2003. Three sputum specimens were microscopically examined of persons reporting a prolonged cough (≥ 3 weeks). Case detection was assessed by the ratio between notification and prevalence.

**Results:**

Of 68,946 included persons (95% response), 1,298 (1.9% 95%CI 1.8–2.2) reported a prolonged cough. Of these, eighteen were sputum smear-positive of whom two had had anti-tuberculosis treatment. The prevalence of new smear-positive tuberculosis was 27/100,000 (95%CI 11–44/100,000) and the notification rate was 44/100,000 among persons ≥ 15 years. The estimated case detection rate was 76%.

**Conclusion:**

Low tuberculosis notification in this mountainous setting is probably a true reflection of low tuberculosis incidence. Possible causes for low incidence in mountainous areas include low transmission rates or altitude-related differences in pathology.

## Background

Tuberculosis is an important cause of morbidity and mortality in low-income countries. Worldwide there were 3.9 million new patients with smear-positive pulmonary tuberculosis in 2003 as estimated by the World Health Organization (WHO) [1].

Studies from Kenya and Mexico suggest that tuberculosis incidence declines with increasing altitude [2,3]. A possible explanation is low incidence at high altitudes, for instance associated with low transmission rates in isolated populations or altitude-related differences in pathology. However, the findings from Kenya and Mexico were based on notification data. Therefore, low case notification could as well be explained by low case detection, e.g. due to limited access of health services.

Vietnam is a high burden country for tuberculosis with a notification rate of new smear-positive pulmonary tuberculosis of 69/100,000 population in 2003 [1]. The National Tuberculosis Control Programme is well established and has nationwide coverage [4]. However, notification rates are considerably lower in mountainous provinces [4] with 26/100,000 population (all ages) in the Central Highlands region in 2003. These low tuberculosis case notification rates in mountainous areas may be due to low incidence or low case detection [4].

A way to clarify this debate is to estimate the case detection rate (i.e. the proportion of incident cases detected by the tuberculosis control programme) by comparing the number of notified tuberculosis cases with the number of undetected infectious tuberculosis patients in the population by a tuberculosis prevalence survey [5]. This study aims to assess the prevalence and case detection of new smear-positive pulmonary tuberculosis among persons with prolonged cough in central mountainous Vietnam.

## Methods

### Study setting

The Central Highlands are at an altitude between 500 meter and 2442 meter above sea level. The population of the Central Highlands was around 4.7 million in 2003 [6]. It is one of the poorest geographic regions in Vietnam and is bordering Laos. HIV prevalence was low at the end of 2004 with 31/100,000 population [7].

### The study

The Central Highlands region consists of four mountainous provinces: Lam Dong, Dac Lac, Gia Lai and Kon Tum. In Lam Dong a survey was carried out in 2002 and therefore excluded from this study. In each of the three remaining provinces four districts were selected with sampling probability proportional to population size. Within each selected district one commune was selected by simple random sampling. The sample size was based on the survey in Lam Dong that had shown a prevalence of 62/100,000 population (Hoa, unpublished observations), with a notification rate of 36/100,000 adult population. Based on desired widths of the confidence intervals of 35, 45 and 50/100,000 population for Dac Lac, Gia Lai and Kon Tum, the sample sizes were calculated at 19,430; 11,754 and 9,520 persons, respectively.

The Research board of the National Hospital for Tuberculosis and Respiratory Diseases in Hanoi gave scientific and ethical clearance to implement the study.

A pre-coded structured questionnaire included demographic variables (age, sex, ethnicity), symptoms experienced within three days prior to the interview, duration of cough, previous diagnosis of tuberculosis, health care provider who had made the diagnosis, date of start and completion of tuberculosis treatment and validated questions on smoking behavior.

The survey was carried out in November and December 2003. Excluded were subjects with permanent or temporary residence outside the district, i.e. living less than 3 months in the district. Trained commune health workers visited house-to-house all persons 15 years or older and listed those who reported cough of more than three weeks (tuberculosis suspects) [8]. Listed persons were invited to come for examination to the commune health center. Provincial health staff interviewed the tuberculosis suspects and collected three sputum specimens (one early morning and two on the spot). Sputum smear specimens were microscopically examined using the Ziehl Neelsen staining method. All positive slides and 10% of negative slides were randomly rechecked by the provincial tuberculosis laboratory.

Notification data of new smear-positive tuberculosis patients in 2003 were obtained from the recording and reporting system of the National Tuberculosis Programme, Vietnam.

Province specific population estimates by age and sex of 2003 [6] were used to calculate the population denominators of 15 years and older. It was assumed that the surveyed population resembled the age and sex structure of the province.

### Definitions

A tuberculosis suspect was defined as a person with a reported cough for 20 days or more, or with reported cough of unspecified duration. A case was defined as smear-positive if at least two smears of two separate sputum specimens were positive. Patients were classified as new who had never been treated with anti-tuberculosis drugs, or treated for no longer than 1 month [8].

### Data analysis

Data were entered using Epi Info version 6 and 10% of forms were randomly re-entered. Inconsistencies were checked against raw data, only few errors were found.

Analyses were performed using Microsoft Excel v2002 (Microsoft, Seattle WA, USA) and Stata/SE V8.0 (Stata Corp., College Station Tx, USA).

The percentage cough and the tuberculosis prevalence, with their 95% confidence interval (95%CI) were weighed inversely proportional to the individual sampling probability [9].

Tuberculosis incidence cannot be measured in a single cross-sectional survey. Prevalence, however, can be measured directly in a cross-sectional survey and be used to estimate the patient diagnostic rate (PDR) [5]. The PDR is the rate at which prevalent cases are detected by a control programme and can be used to derive an estimate of the case detection rate [5]. We have used this indicator to assess tuberculosis case detection.

The PDR was calculated as the number of notified cases per 100,000 population per year (pyr) divided by the prevalence per 100,000 population. The corresponding case detection rate (CDR) as proposed by Dye [10] was calculated using the formula: CDR = PDR pyr/(PDR +0.5) pyr [5].

## Results

The survey included 68,946 persons. Prolonged cough was reported by 1,298 (2.0%) persons (table 1) and the duration of cough was unknown for 114 (8.8%) persons. Sputum specimens were examined of 95% of persons with prolonged cough. Interview data were available for all persons with examined sputum specimen.

**Table 1:** Characteristics of the study population and patient diagnostic rate in the Central Highlands in 2003

	*Province*			*Total*
	Dac Lac	Gia Lai	Kon Tum	
Altitude (meter above sea level)	500–800 [24]	800–900 [25]	550–700 [26]	

Survey				
Population	27,908	23,511	17,527	68,946
Prolonged cough	352	507	439	1,298
% cough*	1.7 (0.8–2.5)	2.5 (1.8–3.2)	2.9 (2.1–3.7)	2.0 (1.8–2.2)
Number of new smear positive tuberculosis cases	4	5	7	16
Prevalence* (/100,000 population) (95%CI)	24 (1–48)	31 (18–45)	46 (1–112)	27 (11–44)
				
Notification				
Population (≥ 15 years)	1,249,057	670,735	213,339	2,133,131
Number of notified new smear- positive tuberculosis cases	515	266	153	934
Notification rate (≥ 15 years) (/100,000 population)	41	40	72	44
				
Patient diagnostic rate	1.7 (0.85–41)	1.3 (0.88–2.2)	1.6 (0.64–72)	1.6 (1.0–4.0)

* weighing inversely proportional to the individual sampling probability

Eighteen persons had sputum smear-positive slides of whom none were on anti-tuberculosis treatment at time of the interview and two had had treatment previously. Sixteen persons were classified as new smear-positive tuberculosis cases. The duration of cough in days was not reported for six persons in Kon Tum. The median duration of cough of the new smear-positive tuberculosis cases was 28 days (inter-quartile range 22–45 days); two cases reported cough for longer than 8 weeks.

The notification rates of new smear-positive pulmonary tuberculosis in 2003 for the total population (including children) were 25, 24 and 42/100,000 population pyr for Dac Lac, Gia Lai and Kon Tum, respectively. The overall notification rate of the three provinces for the population 15 years and older was 44/100,000 population pyr (table 1).

The overall prevalence of new smear-positive tuberculosis was 27/100,000 population pyr (table 1) and the overall PDR was 1.6 per person-year (table 1), corresponding to CDR (as used by the WHO) of 76% (95%CI 67–89%). The prevalence and notification rate among adults were comparable in Dac Lac and Gia Lai, but higher in Kon Tum (table 1).

The prevalence of new smear-positive pulmonary tuberculosis did not differ between men and women. The proportion of people with prolonged cough, the number of prevalent new smear-positive tuberculosis patients and the notification rates increased with age (table 2). The prevalence was higher among people belonging to ethnic minorities compared to people belonging to Vietnam's ethnic majority group, the Kinh (table 2).

**Table 2:** Demographic characteristics and tuberculosis prevalence of the study population in the Central Highlands in 2003

	*Population N*	Prolonged cough* N (%, 95%CI)	*New smear-positive tuberculosis N*	Prevalence* (/100,000 population) (95%CI)	*Notification (/100,000 population pyr)*
Total	68,946	1,298 (1.9%, 95%CI 1.8–2.0%)	16	23 (13–38)	44
Sex					
Men	34,809	721 (2.1%, 95%CI 1.9–2.2%)	9	26 (12–49)	62
Women	34,135	559 (1.6%, 95%CI 1.5–1.8%)	6	18 (6–38)	25
Unknown		18	1		
Age (years)					
15 – 34	40,480	190 (0.47%, 95%CI 0.41–0.54%)	0	0 (0–9)	22
35 – 54	21,165	410 (1.9 %, 95%CI 1.8 – 2.1 %)	6	28 (10–62)	85
55+	7,301	696 (9.5 %, 95%CI 8.9 – 10 %)	10	137 (66–252)	125
Unknown		2	0		
Ethnicity					
Kinh	36,862	488 (1.3%, 95%CI 1.2–1.4%)	2	5 (0–20)	.
Minority group	32,084	796 (2.5%, 95%CI 2.3–2.7%)	13	41 (22–69)	.
Unknown		14	1		

*Unweighted estimate

## Discussion

This study showed a low prevalence of 27/100,000 population of adult new smear-positive pulmonary tuberculosis in three mountainous provinces in 2003 in Vietnam. The estimated case detection rate (CDR) was 76%. In tuberculosis control a CDR > 70% is considered as high and in the same range as the estimated CDR for Vietnam at large of 86% [1], suggesting that the low notification rate reflects low tuberculosis incidence rather than low case detection in this mountainous area. Moreover, tuberculosis prevalence in the Central Highlands was significantly lower than that in a rural district in northern Vietnam, where prevalence was 70/100,000 in 2000 [11] and than in Hanoi, where prevalence was 146/100,000 in 2003/2004 [12]. Therefore, low case detection does not appear to be the main explanation for the low case notification in this area.

These findings are consistent with findings from Kenya and Mexico where notification rates decreased steeply with increasing altitude [2,3]. In Peru, the prevalence of latent tuberculosis infection was significantly lower in high-altitude villages than in villages at sea level [13], or than in high-altitude urban communities [14]. Possible explanations for a lower incidence in this mountainous area in Vietnam and possibly in other high altitude areas may be limited tuberculosis transmission in these remote, sparsely populated areas, or a biological effect associated with high altitude. This includes differences in climate [15] and, for instance lower oxygen tension at high altitude may inhibit the growth rate of mycobacterium tuberculosis in the lung [16].

Other possible causes for low tuberculosis notification and probably low tuberculosis incidence in the Central Highlands include a lower HIV prevalence than in Vietnam in general. To test this hypothesis representative data on HIV prevalence are needed, but are not available for the Central Highlands. Low notification rates may also be due to a younger population in the Central Highlands, as smear-positive pulmonary tuberculosis among persons < 15 is rare. The proportion of the population < 15 years was 30% in Vietnam and 40% in the Central Highlands in 2003. However, notification rates among the adult population were also lower compared to Vietnam in general (Table 1), and therefore a different age structure is insufficient to explain the findings.

Our study has limitations. Underestimation of the number of tuberculosis cases may have occurred due to the way subjects were selected in the survey for tuberculosis diagnosis (by screening for prolonged cough), and to the diagnostic method (sputum smear microscopy). The sensitivity of prolonged cough for detecting smear-positive pulmonary tuberculosis is around 75% [17]. In this study 2.0% of adults reported a cough, which is higher than 1.6% of adults in rural northern Vietnam [18]. Chest radiography could have detected more persons for sputum examination, and culture is a more sensitive method for diagnosing tuberculosis. However, the criteria for defining a suspect and a new smear-positive case were the same in this study as used by the National Tuberculosis Programme Vietnam for routine case detection. The PDR measures the effectiveness of the tuberculosis programme to detect infectious tuberculosis cases and in this study the same criteria were used for the numerator and denominator. Due to incomplete sensitivity the estimates obtained probably underestimate overall prevalence. However, they probably provide a fairly realistic estimate of the prevalence of infectious tuberculosis eligible for diagnosis and treatment in the National Tuberculosis Programme.

The CDR is the proportion of incident cases detected by the National Tuberculosis Programme. The CDR as estimated by WHO is based on the annual risk of tuberculosis infection (ARTI) assuming a fixed ratio between ARTI and tuberculosis incidence [19]. However, this ratio between ARTI and incidence may differ by setting. We used tuberculosis prevalence to estimate the CDR. Therefore, the CDR estimated for Vietnam in general and for the Central Highlands are based on two different methods. An actual lower CDR for Vietnam in general than estimated by WHO (82%) would result in a higher tuberculosis incidence at lower altitudes and therefore strengthen the suggestion that tuberculosis incidence is lower in mountainous areas. An actual higher CDR for Vietnam of 100% would still result in a higher incidence in Vietnam in general than in the Central Highlands.

The translation of the PDR to the estimated CDR depends on assumptions about the duration of disease until death or self-cure in the absence of treatment [5]. This was 2 years in the pre-chemotherapy era [20-23], hence this parameter is used in our calculations of the CDR as well as in the models used by WHO for its estimate of the CDR [5,10]. If this duration of disease is varied from 1 to 3 years, the minimum and maximum CDR would be 62% and 100%. Since the case notification rate in the Central Highlands is almost 3 times lower than in the country at large, even the minimum estimate of the CDR suggests that this mainly reflects lower tuberculosis incidence.

## Conclusion

This study demonstrated low tuberculosis prevalence and a high tuberculosis case detection rate, suggesting low tuberculosis incidence rather than low case detection in this mountainous setting. It is consistent with the findings in other countries where tuberculosis notification decreased with increasing altitude, and merits further studies into causes for low tuberculosis incidence in mountainous areas.

## Abbreviations

CDR case detection rate

PDR Patient diagnostic rate

pyr per year

WHO World Health Organization

## Competing interests

The author(s) declare that they have no competing interests.

## Authors' contributions

DNS and NVC are responsible for study conception. MV, NBH, FC, MB are responsible for the study design. MV and NBH and contributed to the acquisition of the data. NBH planned and conducted the study. MV drafted the first version of the manuscript. All authors have been involved in revising it critically for important intellectual content. All authors read and approved the final manuscript.

## Pre-publication history

The pre-publication history for this paper can be accessed here:


